# Supplementary material for: Live imaging and multimodal profiling reveal transdifferentiation of a cochlear supporting cell subpopulation upon Notch inhibition
Source: Sci Adv. 2026 Jun 17;12(25):eaed3887. doi: 10.1126/sciadv.aed3887 (PMC13274614; doi:10.1126/sciadv.aed3887)
Supplement: Supplementary file 1 — Figs. S1 to S6 Table S1 Legends for tables S2 to S6 Legends for movies S1 to S3 [file sciadv.aed3887_sm.pdf]

Science Advances
AAAS

Supplementary Materials for

Live imaging and multimodal profiling reveal transdifferentiation of a cochlear supporting cell subpopulation upon Notch inhibition

Lama Khalaily *et al.*

Corresponding author: Karen B. Avraham, karena@tauex.tau.ac.il

Sci. Adv. **12**, eaed3887 (2026)
DOI: 10.1126/sciadv.aed3887

The PDF file includes:

Figs. S1 to S6
Table S1
Legends for tables S2 to S6
Legends for movies S1 to S3

Other Supplementary Material for this manuscript includes the following:

Tables S2 to S6
Movies S1 to S3

Supplementary Figures

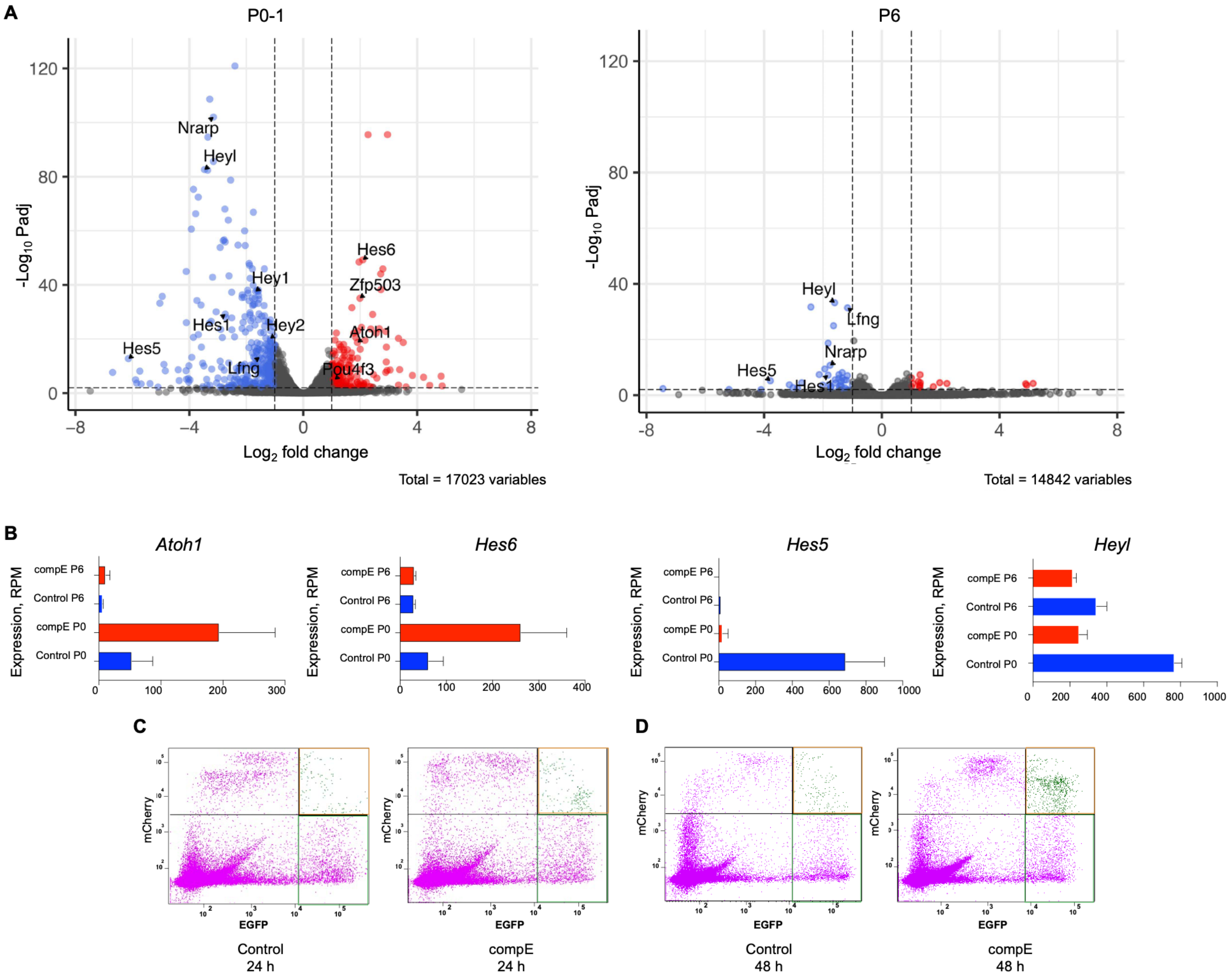

Fig. S1. RNA-seq analysis validated stage-dependent changes in Notch target gene expression following Notch inhibition from supporting cells. (**A**) Volcano plots showing differentially expressed genes upon Notch inhibition with compound E (compE) (2 µM) compared to DMSO control in cochlear samples at P0-1 (left panel) and P6-7 (right panel). At P0-1, 176 genes were significantly upregulated (red dots) and 346 genes were significantly downregulated (blue dots). In contrast, at P6-7, only 15 genes were significantly upregulated, and 40 genes were downregulated, indicating a reduced transcriptional response to Notch inhibition at older stages (n = 6-8 cochleae). (**B**) Expression of key hair cell (HC) and supporting cell (SC) markers across developmental stages and treatment conditions. HC markers *Atoh1 and Hes6* show increased expression upon Notch inhibition at P0-1, but this response is diminished at P6-7, correlating with restricted transdifferentiation potential at older stages. SC markers *Hey1 and Hes5* show a decrease in expression, indicating a shift toward HC fate. (**C, D**) Representative FACS profile for sorting of P0 $Lfng^{EGFP}Atoh1^{mCherry}$ cochleae after culture with DMSO or compound E for both 24 h and 48 h after culturing, used for sorting $EGFP^{+}$ only (green box) and $EGFP^{+}mCherry^{+}$ populations to quantify percentage of double positive cells (orange box). The double positive cells are found in both control and treatment Notch inhibition, but the number increased after compound E treatment, indicating the transdifferentiation events.

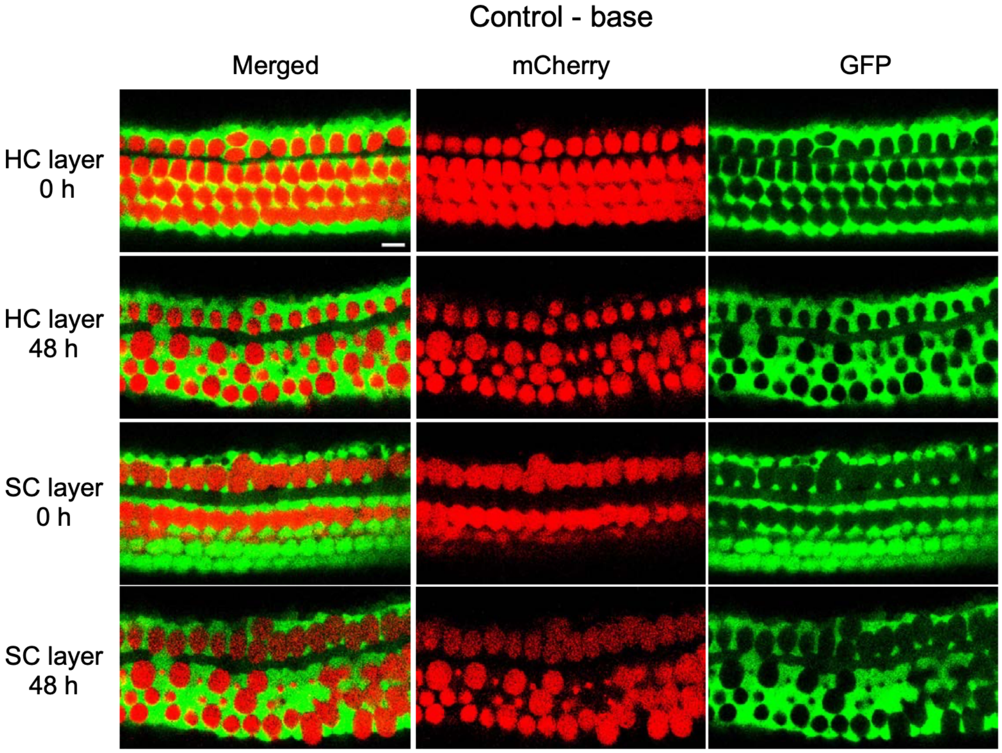

Fig. S2. The precise pattern and layer organization in base treated with Notch inhibition. Filmstrips from $Lfng^{EGFP}Atoh1^{mCherry}$ cochlear explants showing compound E-treated samples on base region. Images represent the HC and SC layers at 0 h and 48 h.

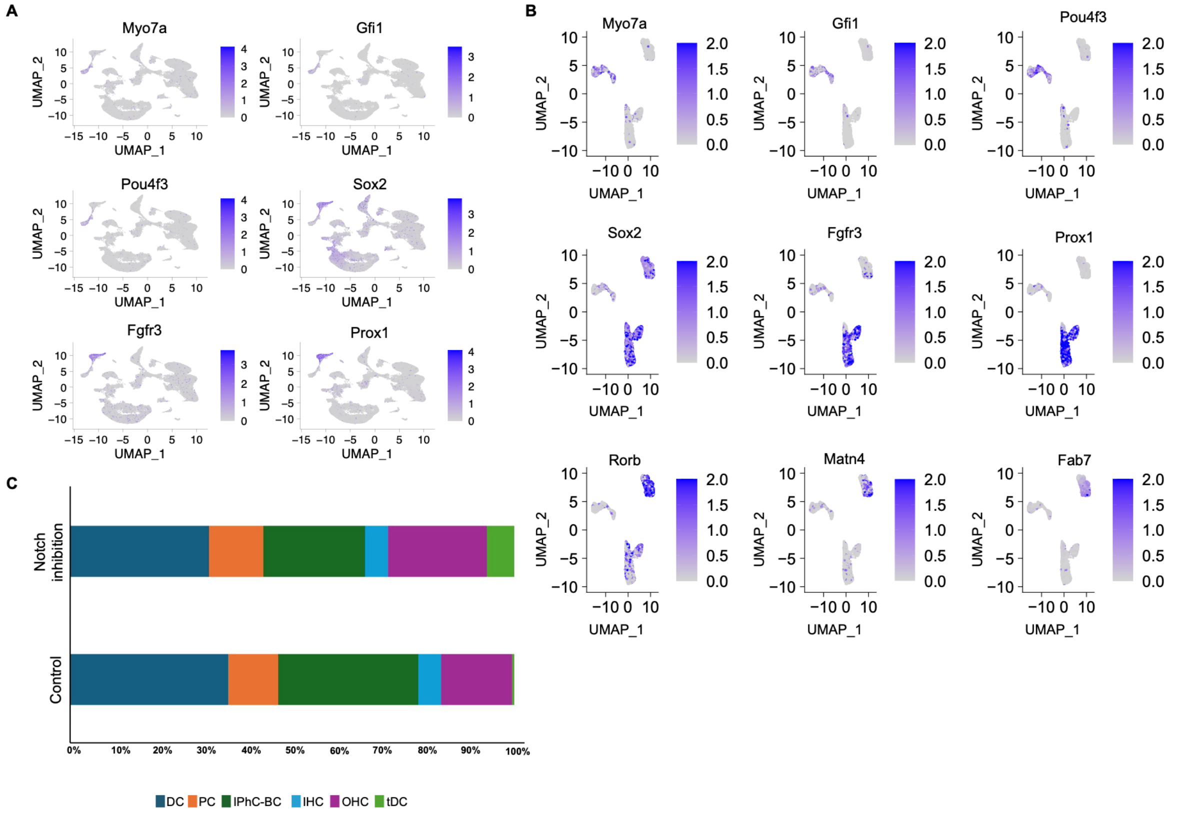

Fig. S3. Marker gene expression and cluster distribution following Notch inhibition. (**A**) Cluster annotation for expression of canonical marker genes across the UMAP projection used for identifying major cochlear cell populations. HC clusters were marked by *Myo7a*, *Gfi1*, and *Pou4f3*; DC and PC clusters by *Prox1* and *Fgfr3*; and IPhC-BC clusters by *Sox2*, consistent with their known molecular identities.(**B**) Marker gene projections onto the subcluster-level UMAP confirmed these assignments: tDCs expressed early HC-specific genes (*Myo7a*, *Pou4f3*, *Gfi1*), whereas stable DCs retained SC identity genes (*Sox2*, *Prox1*, *Fgfr3*). PCs expressed *Sox2*, *Fgfr3*, and *Prox1*, while IPhCs-BCs were characterized by *Sox2*, *Matn4*, *Fabp7*, and *Rorb*. (**C**) Proportions of cell per cluster in control versus Notch inhibition (compound E) samples shows enrichment of tDCs following Notch inhibition.

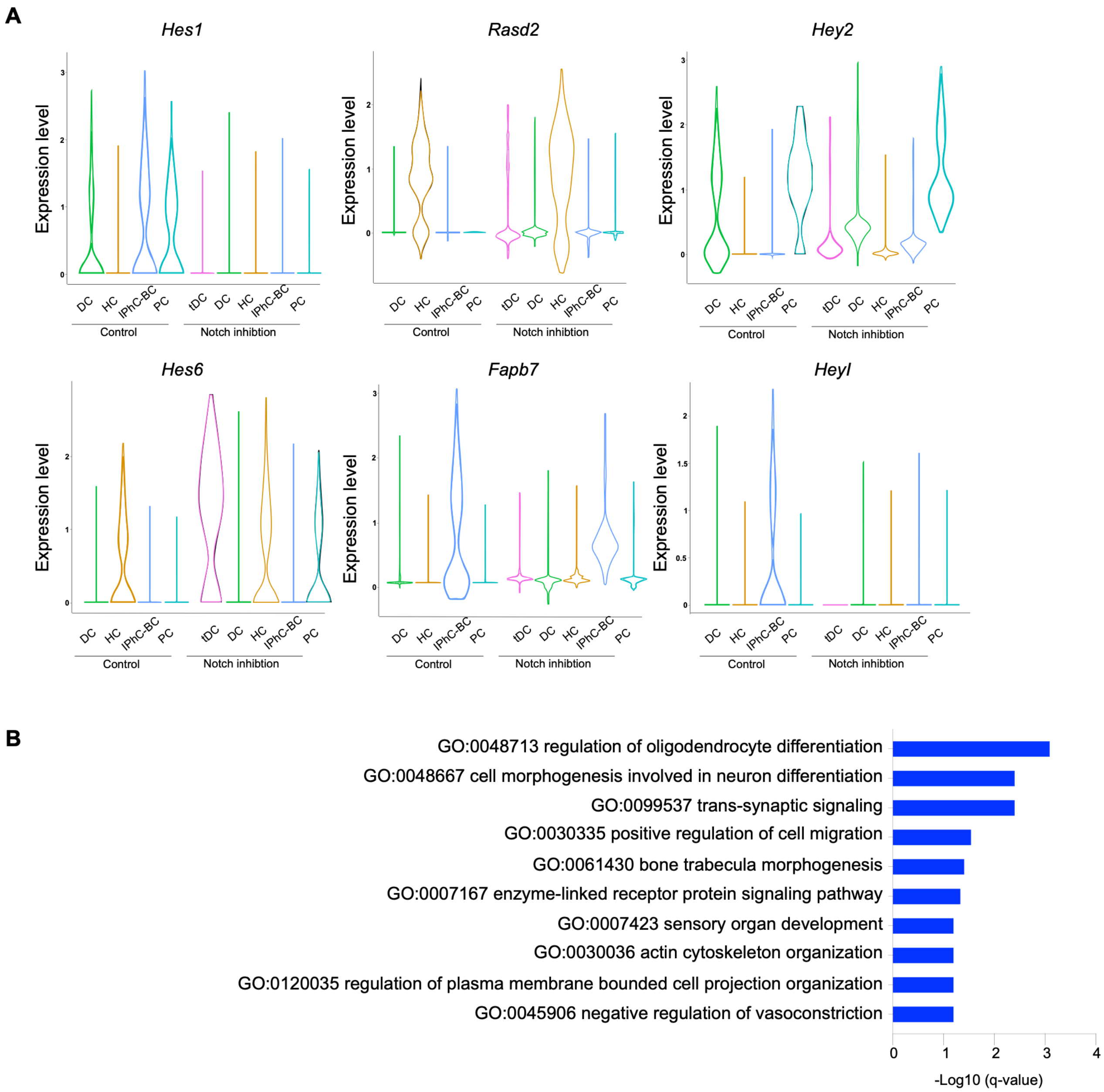

Fig. S4. Gene expression changes and gene ontology for down-regulated genes. (**A**) Gene expression changes shown by violin plots of canonical genes, *Hes1*, *Rasd1*, *Hey2*, *Hes6, Fabp7* and *Heyl*, and across cochlear cells from control to compound E-treated samples. Notch inhibition led to down regulation of *Hes1*, *Hey2*, *Fabp7* and *Heyl*, across all SC populations, while *Hes6* and *Rasd1* was selectively up regulated in tDCs, consistent with initiation of HC-associated transcriptional programs. *Hey2* expression remained high in PCs, and *Fabp7* persisted in IPhCs, indicating incomplete repression in these subtypes**.** (**B**) Gene Ontology enrichment analysis of down-regulated genes in tDCs identified processes related to cell morphogenesis, cytoskeletal reorganization, and migration, reflecting a transition from a stable epithelial to a motile, remodeling state.

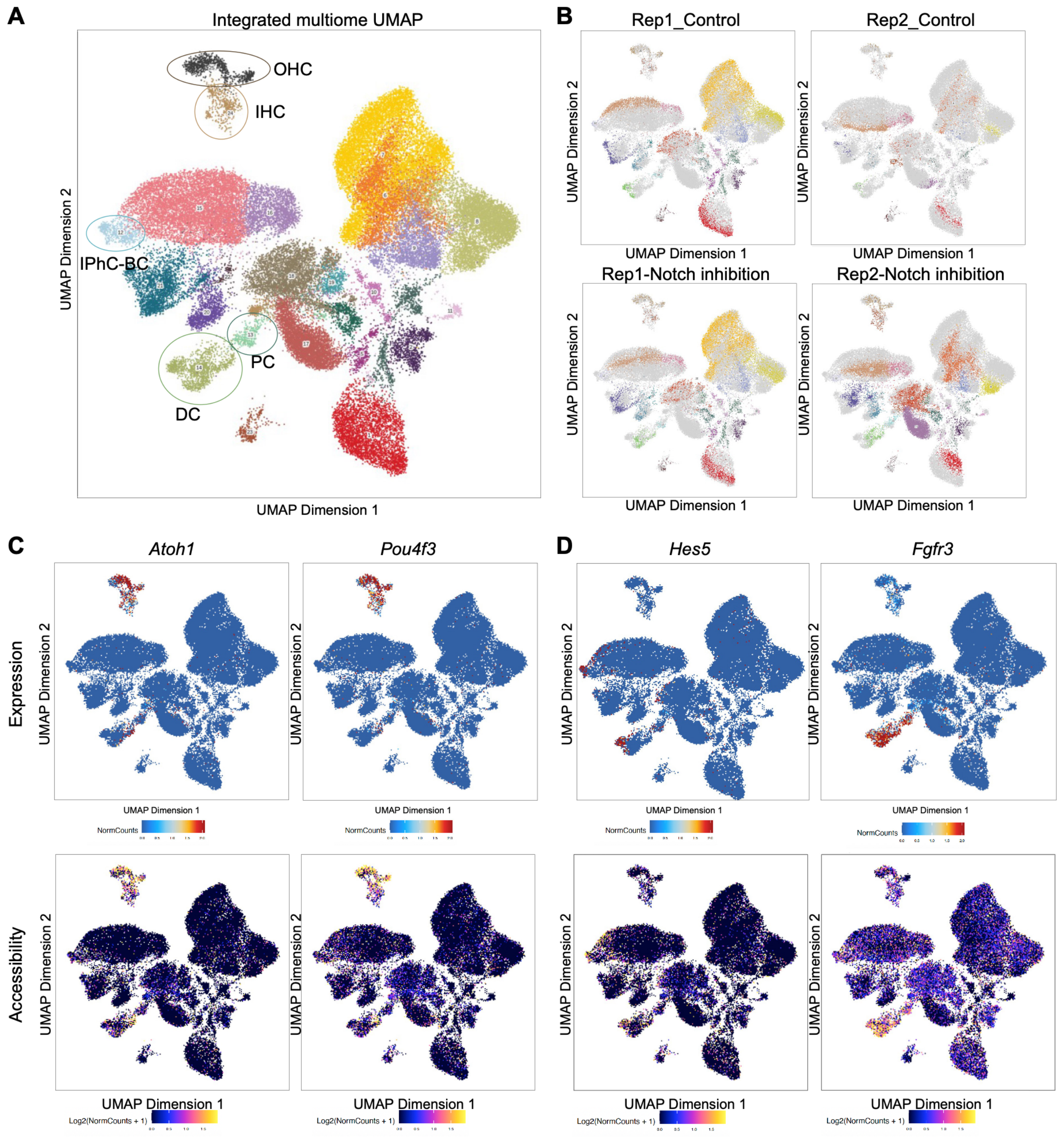

Fig. S5. Multi-ome integration, sample origin, and concordance between gene expression and chromatin accessibility. (**A**) UMAP projection generated from integrated sc-RNA and ATAC multi-ome data, colored by cluster identity and annotated with major cochlear cell populations. (**B**) The same UMAP coordinates colored by sample origin, showing cells from DMSO controls (top row) and compound E treatment (bottom row) across two biological replicates. (**C**) Representative feature plots showing concordance between gene expression (top) and chromatin accessibility (bottom) for HC-associated genes (*Atoh1, Pou4f3*). Accessibility patterns closely mirror transcriptional enrichment in HC clusters. (**D**) Feature plots of SC-associated genes. Both *Hes5*, a canonical Notch target, and *Fgfr3*, a SC marker, show enriched expression and accessibility within SC clusters, consistent with their established roles in SC identity.

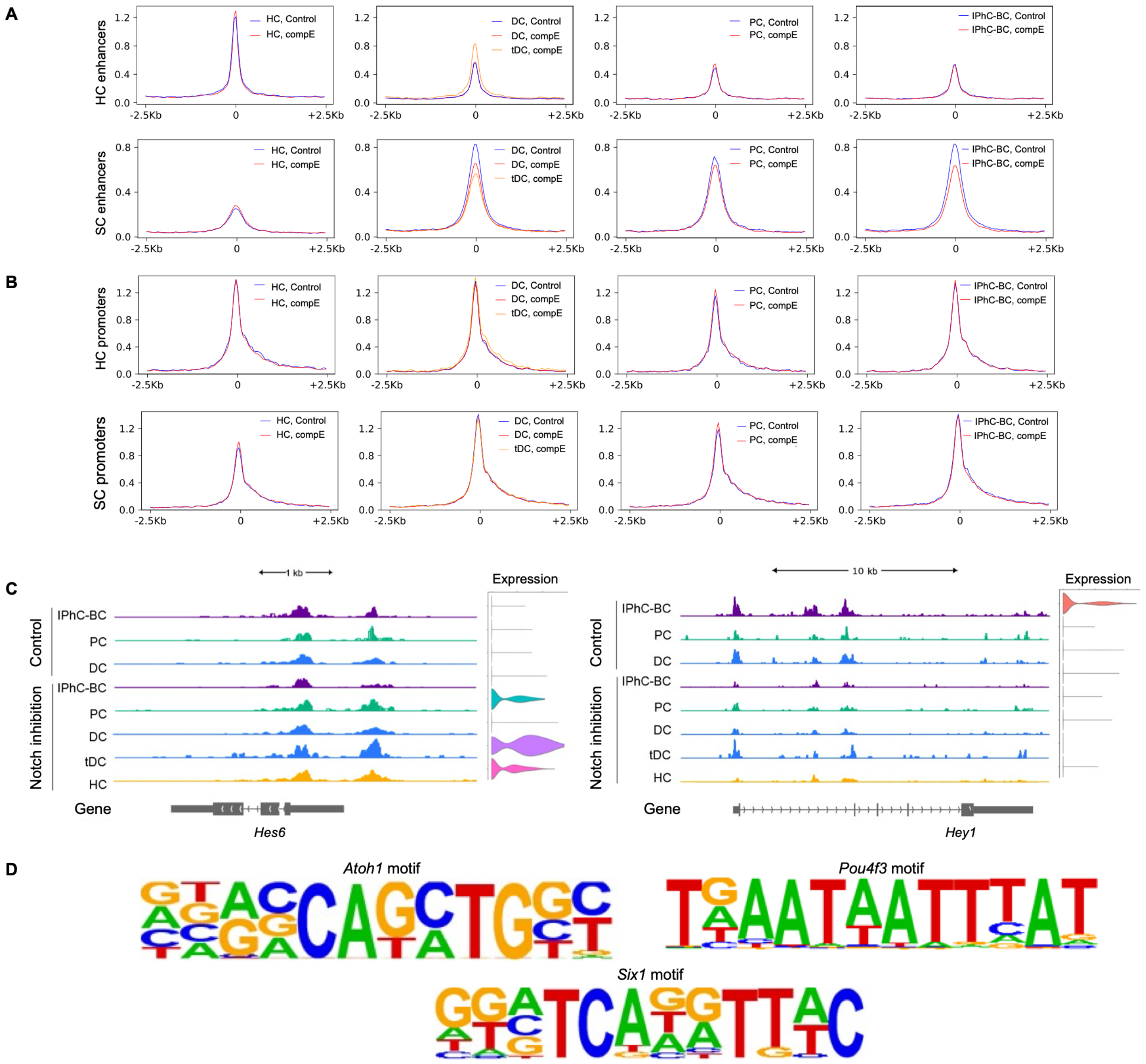

Fig. S6. Chromatin accessibility remodeling and HC motif enrichment in tDCs following Notch inhibition. (**A-B**) Aggregate accessibility plots for enhancer and promoter regions of HC and SC. (**A**) Accessibility profiles centered on transcription factor binding sites within HC-specific (top) and SC-specific (bottom) enhancers. tDCs showed a striking gain of accessibility at HC-specific enhancers compared with DCs and PCs, while SC enhancer accessibility showed some lower accessibility compared to control DCs**.** (**B**) Aggregate accessibility plots for promoter regions of HC and SC. Accessibility centered on transcription start sites of HC-specific (top) and SC-specific (bottom) promoters. Only a small subset of promoters displayed tDC-specific accessibility changes (summarized in table S4). (**C**) Representative ATAC-seq signal tracks showing accessibility profiles at *Hes6* and *Heyl* across cochlear cell types from control and **c**ompound E-treated samples. Each track represents aggregated accessibility from IPhC-BC, PC, DC, tDCs, and HC with their expression level. Notch inhibition maintained stable accessibility for *Hes6* and *Heyl* across all conditions. (**D**) Motif analysis of tDC-HC shared HC enhancers revealed significant enrichment for *Atoh1*, *Pou4f3*, and *Six1* motifs, indicating that tDCs engage canonical HC transcriptional regulators through enhancer activation.

Supplementary Tables

Table S1. Overview of bulk RNA-seq datasets from P0 and P6 cochlear explants under Notch inhibition (compound E, compE) and control conditions (DMSO) (related to Fig. S1A).

Sample	Total reads, mln	Uniquely mapped, mln	Uniquely mapped, %
P0_compE_rep1	65.1	54.5	83.7
P0_compE_rep2	26.9	22.0	81.7
P0_compE_rep3	48.3	39.7	82.2
P0_compE_rep4	44.4	36.6	82.4
P0_DMSO_rep1	37.9	31.2	82.4
P0_DMSO_rep2	54.6	45.1	82.6
P0_DMSO_rep3	44.5	36.0	80.9
P0_DMSO_rep4	42.0	34.5	82.0
P6_compE_rep1	32.4	27.6	85.0
P6_compE_rep2	35.4	30.9	87.3
P6_compE_rep3	35.8	30.9	86.3
P6_DMSO_rep1	30.4	25.9	85.2
P6_DMSO_rep2	34.8	29.9	85.9
P6_DMSO_rep3	54.4	45.9	84.4

Table S2. Quantification and fate tracking of GFP^{+}mCherry^{+} double-positive cells during live imaging in compound E-treated explants (related to Fig. 2).

Table S3. Gene Ontology enrichment (Biological Process) and annotation for up-regulated and down-regulated genes in tDCs compound E vs. DC-DMSO.

Table S4. Differential promoter accessibility analysis for tDCs compared to control DCs (DESeq2 results for promoter-associated peaks).

Table S5. Summary of motif enrichment analysis in differentially accessible regions (HOMER: de novo + known motifs).

Table S6. Pseudobulk RNA-seq differential expression analysis (DESeq2 results).

Supplementary Movies

Movie S1. *Lfng*^{EGFP};*Atoh1*^{mCherry} cochlear explant from the apex region treated with compound E, showing the emergence of GFP^{+}mCherry^{+} double-positive cells of the OHC region. Scale bar: 10 μm.

Movie S2. *Lfng*^{EGFP};*Atoh1*^{mCherry} cochlear explant from the apex region treated with DMSO (control), showing stable tissue organization and absence of new mCherry^{+} cells. Scale bar: 10 μm.

Movie S3. *Lfng*^{EGFP};*Atoh1*^{mCherry} cochlear explant from the basal region treated with compound E, showing no detectable appearance of GFP^{+}mCherry^{+} double-positive cells, indicating minimal responsiveness to Notch inhibition at the base. Scale bar: 10 μm.
